# Genotoxicity of charged particles of importance in space flight using murine kidney epithelial cells

**DOI:** 10.1093/jrr/rrt191

**Published:** 2014-03

**Authors:** Amy Kronenberg, Stacey Gauny, Gianfranco Grossi, Cristian Dan, Dmytro Grygoryev, Mitchell Turker

**Affiliations:** 1 Life Sciences Division, Lawrence Berkeley National Laboratory, Berkeley, CA 94720, USA; 2 Universita Federico II, Naples, Italy; 3 Oregon Health & Science University, Portland, OR, USA

**Keywords:** charged particles, heavy ions, mutation, cell killing, epithelium

## Abstract

Ionizing radiation presents significant challenges for human space flight including an increased cancer risk. High-energy heavy ions in the galactic cosmic radiation can produce qualitative and quantitative differences in biological effects when compared with sparsely ionizing radiations. Mutations are induced by charged particle exposure and are integral to the formation and/or progression of human cancers. Most cancer-associated mutations occur on autosomal chromosomes, and most solid cancers occur in epithelial tissues. Here, a combined *in vitro/in vivo* approach was used to evaluate cell killing and the induction of mutations at a model autosomal locus, *Aprt*, in mouse kidney epithelium. For *in vitro* exposures, *Aprt* heterozygous kidney cells (clones 1a, 4a or 6a) were used from C57BL/6×DBA/2 mice. Additional experiments were performed using whole body irradiation of mice with the same genotype. Both males and females were irradiated in approximately equal numbers. Irradiations were performed at the NASA Space Radiation Laboratories at Brookhaven National Laboratory. For *in vitro* studies, cells from primary kidney clones were irradiated and seeded at limiting dilution immediately post-irradiation to determine the toxicity of the treatment. The irradiated kidney cells were also seeded in mutation assays within 1 week post-irradiation to determine the *Aprt* mutant fraction at the earliest time post-exposure. This work was complemented by studies wherein mice were exposed to the same ions with kidneys harvested several months post-irradiation to determine the residual toxicity and the *Aprt* mutant fraction. Our previous studies focused on sparsely ionizing 1 GeV protons (LET = 0.24 keV/µm) and densely ionizing 1 GeV/amu Fe ions (LET = 151 keV/µm). Our most recent studies have included work with Si ions (240 MeV/amu for *in vitro* studies, LET = 78 keV/µm; 263 MeV/amu initial energy for *in vivo* studies to achieve 78 keV/µm near the midline of the animal) and O ions (250 MeV/amu *in vitro* studies only, LET = 25 keV/µm). Toxicity for the cultured kidney cells *in vitro* follows this pattern: Fe > Si > O > protons when the results are expressed per unit dose. *D*_0_ values were 92 cGy for Fe ions, 103 cGy for Si ions, 192 cGy for O ions and 340 cGy for protons. With regard to the induction of *Aprt* mutations, Fe ions were more mutagenic than protons. Si ions were also quite mutagenic with evidence for a linear dose–response for *Aprt* mutations in kidney cells exposed *in vitro* or in kidneys harvested from mice irradiated several months earlier. These results are consistent with the linear dose–response data obtained previously for *Aprt* mutation induction following Fe ion exposure *in vitro* or *in vivo*, but the results for Si ions differ from the curvilinear dose–response data we recently published following similar exposures to energetic protons [ 1, 2]. Our most recent studies examined the molecular characteristics of Si ion-induced *Aprt* mutants following *in vitro* exposure. A dose of 160 cGy was used to collect 58 *Aprt* kidney cell mutants. Mutational events were classified as follows based on PCR-based analyses of polymorphic markers along mouse chromosome 8: intragenic events, apparent mitotic recombination, interstitial deletions of *Aprt* only, multilocus deletions, discontinuous loss of heterozygosity or whole chromosome loss. The results for this group of mutants will be compared against our previous studies on *Aprt* mutants arising after exposure to sparsely ionizing 1 GeV protons or densely ionizing 1 GeV/amu Fe ions. Additional studies are ongoing to define mutational spectra following Si ion exposure to kidney epithelium *in vivo*.

Clinical Trial Registration number: not applicable.
